# Evolution and Targeting of Myeloid Suppressor Cells in Cancer: A Translational Perspective

**DOI:** 10.3390/cancers14030510

**Published:** 2022-01-20

**Authors:** Augusto Bleve, Francesca Maria Consonni, Chiara Porta, Valentina Garlatti, Antonio Sica

**Affiliations:** 1 Humanitas Clinical and Research Center—IRCCS, 20089 Rozzano, Italy; augusto.bleve@humanitasresearch.it; 2 Department of Pharmaceutical Sciences, Università del Piemonte Orientale “Amedeo Avogadro”, 28100 Novara, Italy; francesca.consonni@uniupo.it (F.M.C.); chiara.porta@uniupo.it (C.P.); valentina.garlatti@uniupo.it (V.G.); 3 Center for Translational Research on Autoimmune & Allergic Diseases (CAAD), Università del Piemonte Orientale “Amedeo Avogadro”, 28100 Novara, Italy

**Keywords:** innate immunity, tumor-associated myeloid cells, tumor-associated macrophages (TAMs), myeloid-derived suppressor cells (MDSCs), tumor microenvironment, cancer immunotherapy

## Abstract

**Simple Summary:**

Immunotherapy is achieving impressive results in the treatment of several cancers. While the main strategies aim to re-invigorate the specific lymphocyte anti-tumor response, many studies underline that altered myeloid cell frequency and functions can dramatically interfere with the responsiveness to cancer therapies. Therefore, many novel strategies targeting TAMs and MDSCs in combination with classical treatments are under continuous evolution at both pre-clinical and clinical levels, showing encouraging results. Herein, we depict a comprehensive overview of myeloid cell generation and function in a cancer setting, and the most relevant strategies for their targeting that are currently in clinical use or under pre-clinical development.

**Abstract:**

In recent years, the immune system has emerged as a critical regulator of tumor development, progression and dissemination. Advanced therapeutic approaches targeting immune cells are currently under clinical use and improvement for the treatment of patients affected by advanced malignancies. Among these, anti-PD1/PD-L1 and anti-CTLA4 immune checkpoint inhibitors (ICIs) are the most effective immunotherapeutic drugs at present. In spite of these advances, great variability in responses to therapy exists among patients, probably due to the heterogeneity of both cancer cells and immune responses, which manifest in diverse forms in the tumor microenvironment (TME). The variability of the immune profile within TME and its prognostic significance largely depend on the frequency of the infiltrating myeloid cells, which often represent the predominant population, characterized by high phenotypic heterogeneity. The generation of heterogeneous myeloid populations endowed with tumor-promoting activities is typically promoted by growing tumors, indicating the sequential levels of myeloid reprogramming as possible antitumor targets. This work reviews the current knowledge on the events governing protumoral myelopoiesis, analyzing the mechanisms that drive the expansion of major myeloid subsets, as well as their functional properties, and highlighting recent translational strategies for clinical developments.

## 1. Introduction

While the functional plasticity of myeloid cells has assumed considerable interest as a potential level of therapeutic intervention in tumors, the mechanisms that drive their protumoral phenotype are only partially elucidated, and research is mainly focused on understanding the intratumoral signals capable of polarizing myeloid cell functions. Nevertheless, recent observations highlighted that the final state of activation and heterogeneity of immune cell responses in cancer bearers is conferred through a multistep process, which includes lineage commitment and expansion of hematopoietic progenitors in the bone marrow (i.e., hematopoiesis), their subsequent mobilization to the periphery and the final recruitment and conditioning in response to signals that operate in the TME [1].

Several inflammatory insults drive “pathological myelopoiesis” [2], including pathogen-associated molecular patterns (PAMPs) and damage-associated molecular patterns (DAMPs) [3], which are sensed by pattern recognition receptors (PRRs) [4]. Innate immune cells activated through PRRs provide the source for cytokines and myelopoietic growth factors, acting on myeloid progenitors. Of relevance, activation of hematopoietic stem cells (HSCs) to persistent low-grade inflammation in cancer or over-activation (i.e., acute infections or sepsis) perpetuates and increases myelopoiesis at the expense of lymphopoiesis, which favors immunosuppression [5]. To complement these mechanisms, new evidence indicates the existence of metabolic gates which control the suppressor myeloid cells in cancer [6], as well as their epigenetic dysfunctions [7]. The gap in the knowledge still present on the mechanisms that drive myelopoietic alterations during tumor growth, as well as on their contribution to tumor development and resistance to anticancer therapies, is becoming increasingly evident. A better understanding of the processes that integrate myelopoietic response, mobilization of myeloid progenitors, their recruitment and functional diversion into the tumor site could herald new advanced therapeutic approaches, also identifying new markers and criteria for personalized therapy.

In accordance with this, increasing evidence shows dysregulated cellular signaling and metabolism in myeloid cell subsets that infiltrate immunologically cold tumors resistant to immune checkpoint inhibitors (ICIs), chemo- and radio-therapy, characterized by a lack in T and NK cell infiltrates, and the accumulation of myeloid-derived suppressor cells (MDSCs), tumor-associated macrophages (TAMs) and tolerogenic dendritic cells (DCs) [8,9].

## 2. Emergency Myelopoiesis

In stationary conditions, hematopoiesis is characterized by strictly controlled and balanced cell transition phases, which allow the conservation of both resident and circulating lymphoid and myeloid cells. In this hierarchically organized process, the apex of the pyramid is occupied by HSCs [10]. HSCs reside primarily in the bone marrow (BM), within a specialized micro-environment defined as the HSC niche. The latter comprises different cellular constituents, which include cells of mesenchymal origin, endothelial cells and HSC progeny, that cooperate to generate effective defenses against pathogens. HSCs are endowed with the ability to control self-renewal and differentiative cell divisions, producing multipotent and lineage-committed progenitors, that in turn can terminally differentiate into both lymphoid and myeloid progenitors [10,11]. Common myeloid progenitors (CMPs), in particular, undergo further selective differentiation, generating granulocyte-macrophage progenitors (GMPs) and monocyte-dendritic cell progenitors (MDPs) [11].

**Figure 1:**
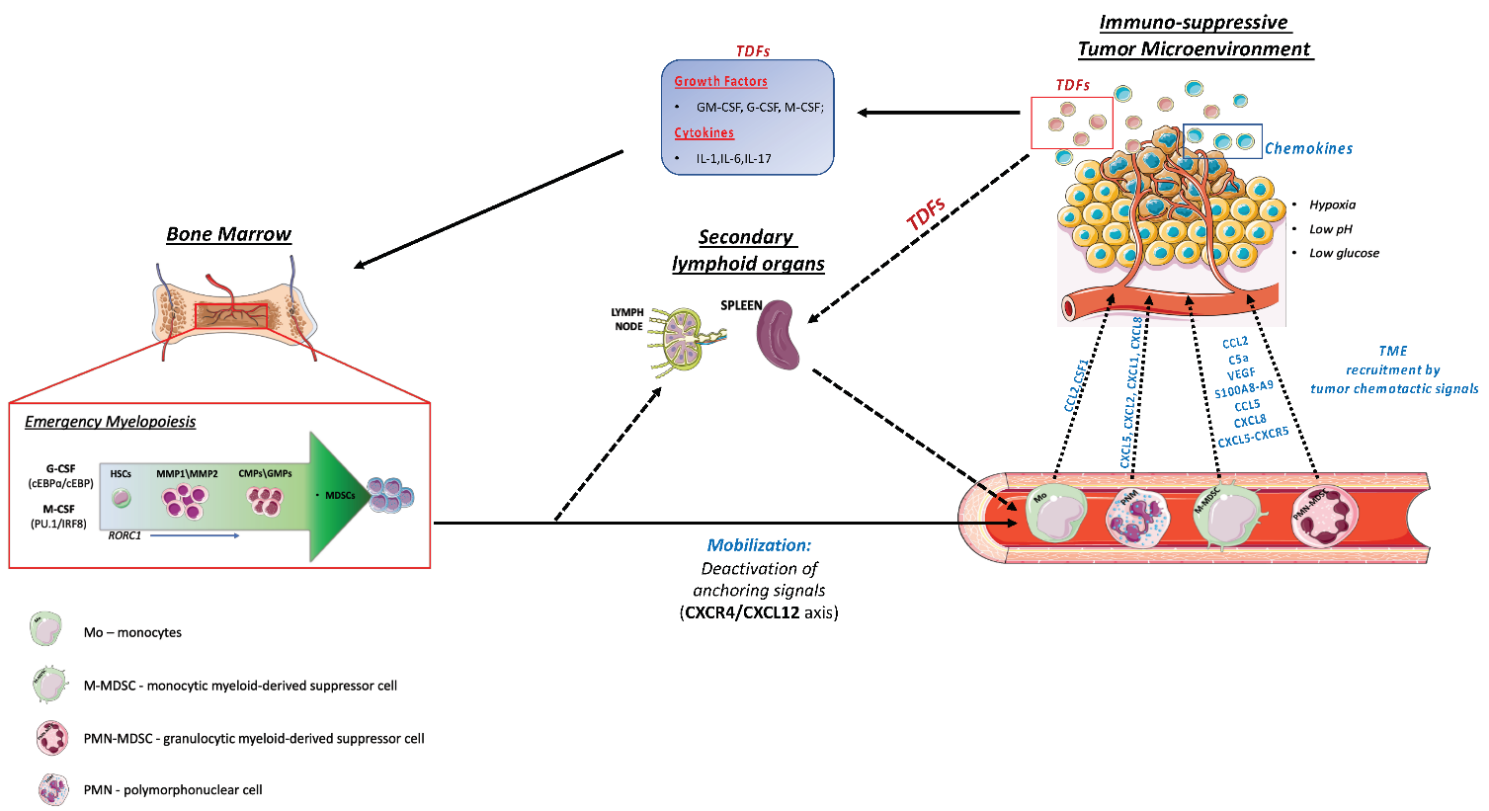
Schematic representation of myelopoiesis in cancer bearers. Tumor-derived factors (TDFs), endowed with myelopoietic activity (i.e., myeloid growth factors/CSFs and cytokines), alter the myelopoietic output, inducing the expansion and mobilization of different subtypes of myeloid suppressor cells. The transcription factor RORC1 is a crucial mediator of this myelopoietic response in emergency conditions. Deactivation of anchoring signals, such as the retention axis CXCR4/CXCL12, induces the mobilization of myeloid cells to the periphery. Once in the circulation, myeloid cells reach the secondary lymphoid organs (i.e., lymph nodes and spleen) and are recruited at the tumor site in response to chemotactic signals. Immunosuppressive cytokines and factors released within the tumor microenvironment (i.e., IL-10, TGFβ, PGE2) and micro physiological conditions (i.e., hypoxia, low glucose levels, low pH) concur to complete the pro-tumoral skewing of myeloid cells. This multistep process establishes local and systemic immunosuppression, which represents a major obstacle for anticancer immunotherapy.

These highly coordinated events are altered by tumors that provide immunological stresses able to amend the hematopoietic output and consequently shape the TME composition, by the recruitment of both mature and immature myeloid precursors characterized by an immunosuppressive potential [1,12]. This pathological expansion of protumoral myeloid cells is defined as “emergency myelopoiesis”. These suppressor populations include monocytic and granulocytic myeloid-derived suppressor cells (M-MDSCs and PMN-MDSCs, respectively), as well as tumor-associated macrophages (TAMs) (Figure 1) and neutrophils (TANs) [1,13]. In addition, the TME is also characterized by the presence of regulatory T cells (Tregs), T-helper 17 cells (Th17), as well as other myeloid cell subsets (not of main interest for this review), including regulatory dendritic cells (DCregs), Tie-2 monocytes and mast cells, which support cancer growth and spread [14,15].

Similarly to infections, cancers promote a switch from homeostatic to emergency myelopoiesis, through the sensing of danger signals from tumor tissue operated by pattern recognition receptors (PRRs) and the consequent activation of downstream signaling pathways that lead to overproduction of myelopoietic cytokines, such as granulocyte (G-CSF) and macrophage colony-stimulating factors (M-CSF) [16], as well as hematopoietic cytokines [1]. Among these, interleukin (IL)-17A induces both G-CSF- and stem-cell-factor-mediated neutrophilia [1].

IL-1 and IL-6 represent additional players. In particular, IL-1 has been found to increase the proliferation and differentiation rate of HSCs through the induction of PU.1 and the consequent upregulation of both the M-CSF/CSF1 (Csf1r) and the GM-CSF (Csf2ra) receptors [17]. Of note, while the TNFα is primarily recognized as an immunostimulatory, anti-tumoral cytokine, an opposing effect of its chronic production emerged, inducing an accumulation of immunosuppressive tumor-promoting myeloid cells [18,19]. Interestingly, aberrant myelopoiesis may be reinforced by chemotherapy (CT), through a mechanism defined as CT-induced inflammation [1,5]. Advances have also been made in understanding the signal transduction pathways involved in the expansion of suppressive myeloid cells. In this regard, a key role of signal transducer and activator of transcription 3 (STAT3) was reported in MDSCs’ expansion and activation [20]. In addition, hematopoietic BM progenitors treated with tumor-derived supernatants exhibited an up-regulation of JAK2-STAT3, increasing MDSCs’ expansion in vitro [20]. C/EBPβ activation critically supports “emergency granulopoiesis”, supporting granulocyte expansion, inhibiting apoptosis and producing a specific set of cytokines, such as IL-6, IL-10 and IL-12 [21]. Evidence obtained from studies on hepatocellular carcinoma (HCC) revealed a possible inter-connection between C/EBPβ and STAT3 in the context of MDSC expansion. In fact, the role of C/EBPβ as co-activator of STAT3 transcriptional activity was also reported [20]. Our group uncovered the role of retinoic acid-related orphan receptor C 1 (RORC1/RORγ), mainly expressed on immature myeloid cells during tumor-related inflammation. Interestingly, RORC1 induces C/EBPβ to sustain myeloid-derived suppressor cell expansion [22]. More recently, a new population of prometastatic TAMs, endowed with high rate of heme catabolism, was shown to be induced by an M-CSF-dependent activation of the transcription factor Nrf2 [23]. Therefore, in order to obtain a more comprehensive understanding of myeloid evolution in cancer carriers, we cannot ignore the integration of multistep mechanisms that alter the commitment of hematopoietic progenitors, their mobilization towards the periphery and the subsequent infiltration of tumors that expose myeloid cells to tumor-derived factors (TDFs) [8].

## 3. Myeloid Cells Mobilization

The prominent accumulation of immune-suppressive myeloid cells (i.e., MDSCs and TAMs) at the tumor site is the result of coordinated events that include the mobilization of myeloid progenitors from the bone marrow to the periphery and their subsequent recruitment at the tumor site [1]. Chemokine/chemokine receptor systems, adhesion molecules (VLA-4, CD44) and cytokines (G-CSF, SCF, FLT3-L) are critical orchestrators of hematopoietic stem and progenitor cells (HSPCs) mobilization. HSPCs’ mobilization is induced clinically or experimentally in animal models by a wide variety of agents, such as cytokines (e.g., G-CSF), chemotherapeutic agents (e.g., cyclophosphamide) and small molecules which interfere with anchoring molecules (e.g., the CXCR4 antagonist AMD3100) [24].

We recently reported that M-CSF, in addition to inducing PU.1-driven myeloid differentiation, has a direct role in controlling the inducible form of nicotinamide phosphoribosyl transferase (iNAMPT) activity, catalyzing NAD biosynthesis [6]. Elevated expression of intracellular NAMPT (iNAMPT) in myeloid progenitors causes negative regulation of the BM retention axis of hematopoietic cells C-X-C motif chemokine receptor 4 (CXCR4), thus undocking these cells and allowing the mobilization of suppressor myeloid cells to the periphery [6]. In agreement with this, iNAMPT inhibition prevents MDSCs mobilization, reactivates specific antitumor immunity and enhances the antitumor activity of ICIs [6]. Various metabolic perturbations contribute to regulating these processes. As examples, glutamine starvation was found to be important for the upregulation of G-CSF and GM-CSF, two well-known facilitators of myelopoiesis and the mobilization of hematopoietic progenitor cells [25], while hypercholesterolemia promoted bone marrow cell mobilization by perturbing the CXCL12/CXCR4 axis [26]. Evidence has been also provided that the hematopoietic-specific phospholipase C (PLC)-β2 lipolytic enzyme promotes the mobilization of hematopoietic stem cells by decreasing their lipid raft-mediated bone marrow retention [27]. Complement component 3a receptor 1 (C3aR1) antagonists restrained neutrophil mobilization, and melanoma-bearing C3aR1-deficient mice had reduced tumor growth and frequency of heme oxygenase 1 (HO-1) expressing monocytic blood precursors of HO-1^+^ TAMs [23,28]. Blocking the C-C motif chemokine ligand 2 (CCL2)/C-C chemokine receptor type 2 (CCR2) pathway is also a rational approach to inhibit the accumulation of M-MDSCs and TAMs in the TME and to limit the mobilization of bone marrow monocytes into the blood stream, since the activation of CCR2 attenuates the CXCR4 anchoring signaling [29,30].

Once in the circulation, monocytes and MDSCs are actively recruited to primary and metastatic tumor sites. This process is regulated by chemokines produced by the tumor [31]. The role of chemokines in the recruitment of myeloid cells to the tumor site influences specific antitumor immunity, metastasis formation and angiogenesis, thus playing a central role in tumor development and clinical response. These events have been extensively examined in recent works and do not represent the focus of the present work [32,33].

## 4. Functional Heterogeneity of Tumor-Associated Myeloid Cells

**Figure 2:**
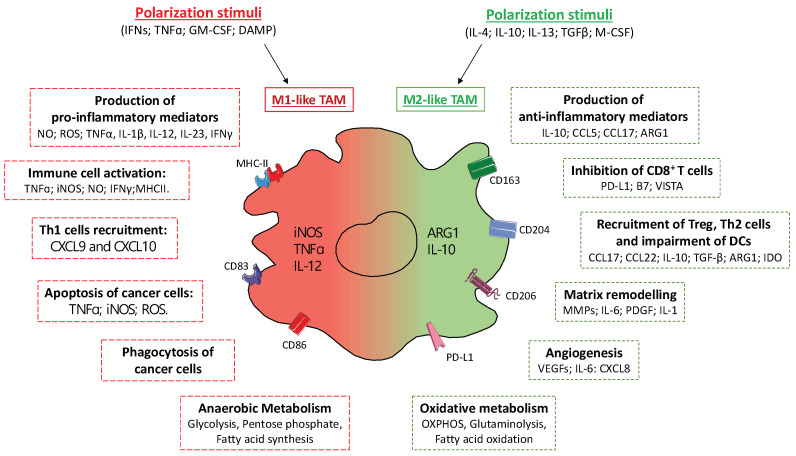
Influence of the polarization state of TAMs in the TME. Immunostimulatory signals (e.g., IFN, TNFα, GM-CSF, DAMP) induce the inflammatory M1 phenotype of TAMs, which through the production of pro-inflammatory cytokines (e.g., TNFα, IL-1β, IL-12, IL-23, IFNγ), reactive oxygen species (ROS) and nitric oxide (NO) promote anticancer conditions. M1 TAMs also support the intratumoral homing and activation of T cells by producing specific chemotactic factors and favoring antigen presentation (e.g., CXCL9, CXCL10, MHC II). Furthermore, they exert tumoricidal activity by means of phagocytosis and direct killing of tumor cells, through cytotoxic mediators (e.g., TNFɑ, NO, ROS). Conversely, M2-like macrophages are activated by immunomodulatory mediators with immunoregulatory properties (e.g., IL-4, IL-10, IL-13, TGFβ, M-CSF) that favor tumor development. Indeed, M2-like TAMs secretespecific anti-inflammatory cytokines (e.g., IL-10) and chemokines (e.g., CCL5, CCL17, CCL22), which inhibit the specific response of T cells while supporting the recruitment of Treg and Th2 lymphocytes. They also support the formation of metastases, producing factors that promote angiogenesis (e.g., VEGFs, IL-6, CXCL8) and matrix remodeling (e.g., MMPs, IL-6, PDGF, IL-1).

**Figure 3:**
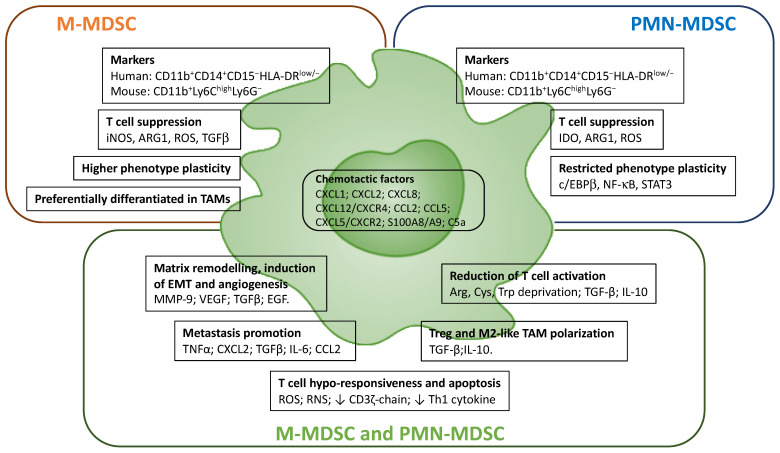
Phenotypic and functional traits of MDSCs. Human and murine markers are reported. MDSCs are recruited to the tumor site through chemotactic factors produced by the tumor. Inside the tumor, MDSCs exert their immunosuppressive functions by inhibiting the antitumor response of T lymphocytes, through various mechanisms. These include the production of immunosuppressive cytokines (e.g., IL-10, TGFβ), the alteration of amino acid metabolism (Arg, arginine; Cys, cysteine; Trp, tryptophan) and peroxynitration of the CD3ζ chain (by peroxynitration). MDSCs can also promote the spread of cancer cells by remodeling the extracellular matrix, promoting EMT and angiogenesis. The figure highlights the specific or shared characteristics of monocytic and granulocytic MDSC subsets. For abbreviations and details, see the text.

Tumor-infiltrating myeloid cells (e.g., macrophages, MDSCs and neutrophils) have a relevant role in establishing the intratumoral immunoprofile, which today represents a widely accepted prognostic index and therapeutic target [34,35]. It is, however, evident that myeloid cells in tumor tissues consist of a dynamic population characterized by considerable plasticity, which includes ontological and functional diverse subsets [36]. This “myeloid heterogeneity” is dictated by both local and systemic stimuli, able to affect specific genetic and epigenetic programs (Figure 2 and Figure 3) [37].

Recently, fate mapping experiments and high dimensional-analytic approaches with single-cell resolution have revealed a picture of the diversity within the myeloid compartment in several cancer types (i.e., lung cancer [38], melanoma [39], renal cancer [40], breast cancer [41], colon cancer [42,43]) dissecting the myeloid population into several distinct clusters, based on differential gene expression, localization in the TME, morphological features and functions [44]. Interestingly, the complexity of myeloid heterogeneity in the TME reflects the variability of therapeutic outcomes observed in cancer patients treated with immunotherapy [45]. Efforts are now converging on deeply understanding on how the activation state, localization and different phenotypes of myeloid cells contribute to the efficacy of anticancer therapies, focusing on the identification of prognostic and predictive markers useful for personalized therapies.

### 4.1. Tumor Associated Macrophages (TAMs)

TAMs include both resident and BM-derived macrophages and represent the most abundant myeloid population in the TME, where they adapt their functions in response to environmental cues [35]. This functional adaptation is oversimplified into the classical (M1) or alternative (M2) macrophage polarization model, with M1 referring to anti-tumorigenic and M2 to pro-tumorigenic macrophages (Figure 2) [46].

In particular, in response to endogenous TLR ligands (i.e., DAMPs) and interferons (IFNs), M1-polarized TAMs express anti-tumorigenic potential by releasing high levels of inflammatory mediators, such as tumor-necrosis factor (TNF)-α, IFNγ, nitric oxide (NO) via inducible NO synthase (iNOS), reactive oxygen species (ROS), and stimulate cytotoxic functions of NK cells and CD8^+^ T cells. Furthermore, their high phagocytic activity combined with the expression of major histocompatibility complex class II (MHCII), costimulatory molecules and T cell-recruiting chemokines (i.e., CXCL9 and CXCL10) make them strong promoters of Th1 responses [46,47]. Conversely, immunosuppressive (IL-10 and TGFβ) and Th2 cytokines (IL-4, IL13), glucocorticoids and intratumor hypoxia promote M2-like TAMs polarization, mainly oriented towards the activation of Th2-type immune responses, extracellular matrix (ECM) remodeling and angiogenesis, mostly through the induction of arginase 1 (ARG1)- and IL-10-mediated and metalloprotease networks, promoting tumorigenesis and development [8].

TAMs are crucial promoters of the neoangiogenic switch in tumors, since their frequency correlates with vascular density in preclinical and human tumors and macrophage depletion strategies have been associated with reduced tumor angiogenesis in different preclinical models. Moreover, in response to hypoxia TAMs upregulate expression of hypoxia inducible factor (HIF)-1α and secretion of angiogenic and lymphangiogenic factors, such as vascular endothelial growth factor A (VEGF-A), VEGF-C, IL-6, CXCL8 [48,49]. A distinct subset of TAMs expressing the TIE2 receptor (TIE2-expressing TAMs, TEMs) has been described to promote angiogenesis through release of proangiogenic and tissue-remodeling factors [50].

M2-like TAMs also contribute to the creation of an immunosuppressive TME via the expression of immune checkpoint ligands, such as PD-L1, PD-L2, B7 and VISTA, which directly inhibit T-cell activation/proliferation and simultaneously decrease essential co-stimulatory molecules, such as CD80 and CD86 [51]. Moreover, TAMs exert indirect effects on adaptive immune responses through the recruitment and activation of Tregs and Th2 cells, via the production of chemokines (i.e., CCL17 and CCL22) and anti-inflammatory molecules (i.e., IL-10, TGF-β, ARG1, IDO), as well as through the inhibition of DCs’ maturation [52]. In the advanced stages of tumor development, M2-like TAMs facilitate the invasive behavior of cancer cell and metastatic progression through the release of various proteases involved in ECM digestion (i.e., members of the MMP and cathepsin families) [53], promoting the evasion of tumor-initiating cells, by expressing mediators of both cancer cell stemness (i.e., IL-6, PDGF, IL-1) [54] and proliferation (i.e., epidermal growth factor/EGF), and facilitating the epithelial-mesenchymal transition (EMT) [55].

In line with these protumor functions, preclinical and clinical data indicate a close relationship between high infiltration of M2-like TAMs and a poor prognosis in most types of tumor, such as pancreatic ductal adenocarcinoma (PDAC), glioblastoma, and bladder cancer [35,55].

On the other hand, a correlation between TAMs’ infiltration and improved cancer-patient survival has also been described in some cases, such as in endometrial cancer [56]. Such discordance can be attributed to the inter- and intra-tumor heterogeneity of TAMs, which may relate to different ontogeny, activation status and intratumor localization [8]. Interestingly, morphometric characterization of human TAMs purified from colorectal liver metastases revealed that large TAMs were associated with a poorer survival rate than small TAMs [42]. In breast cancer, the infiltration of immunosuppressive PD-L1^+^CD38^+^ TAMs is more closely related to estrogen receptor-positive cancerous regions, thus limiting the success of immune checkpoint inhibitors [57].

In spite of the M1 vs. M2 oversimplification, intermediate stages of macrophage polarization can coexist in the same TME, although overall most TAMs closely resemble M2-polarized macrophages [58]. Single-cell sequencing approaches have recently demonstrated the functional heterogeneity of TAMs embedded in different cancers [44,57,59], identifying up to 17 different TAM clusters, each characterized by a specific genetic profile. In liver cancer, Zhang et al. identified a specific subset of TAMs expressing high levels of ferroportin-encoding gene SLC40A1, an iron exporter also involved in the release of pro-inflammatory cytokines, including IL-6, IL-23, and IL-1β, via TLR-mediated signaling and associated with poor prognosis [59]. According to the role of iron metabolism in shaping a protumor TME, we recently described a specific subset of TAMs expressing a high level of heme oxygenase 1 enzyme (HO-1) and reported that the expansion of blood HO-1^+^ monocytes is associated with both increased frequency of HO-1^+^ TAMs and poor prognosis, in both preclinical fibrosarcoma and melanoma models, as well as in stage III-IV melanoma patients [23].

In line with this observation, transcriptional comparison of circulating monocytes derived from healthy subjects and oncological patients with breast and endometrial cancer, as well as characterization of their TAMs, revealed distinct transcriptional signatures, depending on cancer type. Furthermore, for each tumor type, the transcriptional signatures of the TAMs differed from the respective circulating monocytes [60].

TAMs mostly derive from circulating inflammatory monocytes and M-MDSCs, under the guidance of specific chemotactic pathways (i.e., CCL2, CCL5, CXCL12, system of complement) and intratumoral conditions (i.e., hypoxia) [61,62]. Nonetheless, it is now clear that tissue-resident macrophages are also indispensable regulators of the TME and contribute to the TAM population [63]. In malignant glioma, most TAMs derive from resident microglia, rather than circulating monocytes, and actively contribute to tumor progression [64]. In the murine pancreatic ductal adenocarcinoma (PDAC) model, tissue-resident macrophages were found to expand during tumor progression and to promote fibrosis, a major barrier for PDAC therapy, while monocyte-derived TAMs had increased expression of genes associated with immunosuppression and antigen presentation [65].

Additionally, supporting the concept that TAMs harbor ontogenetically and functionally different macrophages, Loyher et al. demonstrated in lung cancer models that monocyte-derived TAMs contribute to tumor spread, while tissue-resident TAMs directly support the proliferation of cancer cells [66]. Moreover, tissue-resident self-renewing CD163^+^TIM4^+^ macrophages in the metastatic omentum are described to provide protective niche for ovarian cancer stem cells and to promote their metastatic spread [67].

### 4.2. Myeloid-Derived Suppressor Cells (MDSCs)

MDSCs are a heterogeneous myeloid cell population characterized by the ability to suppress adaptive antitumor immune responses and directly contribute to both tumor growth and metastatic formation [68]. Currently, as a reflection of their lineage derivation, MDSCs are conventionally divided into two major monocytic and granulocytic subsets, based on their phenotypic and morphological features: monocytic MDSCs (M-MDSCs), which are identified as either human CD11b^+^CD14^+^CD15^−^HLA-DR^low/−^ cells or mouse CD11b^+^Ly6C^high^Ly6G^−^ cells; and polymorphonuclear MDSCs (PMN-MDSCs) characterized as human CD11b^+^CD14^−^CD15^+^HLA-DR^low/−^ cells and mouse CD11b^+^Ly6C^low^Ly6G^+^ cells, respectively [62,69]. However, because MDSCs do not represent an actual subset of myeloid cells but rather a state of activation, understanding the complex nature of MDSCs’ biology remains a great challenge.

In both tumor-bearing mice and cancer patients, MDSCs abundantly expand and accumulate in primary tumors and metastatic lesions, as well as in secondary lymphoid tissues, bone marrow and peripheral blood. Interestingly, frequency of circulating MDSCs is associated with poor clinical outcome in a variety of solid tumors [62,68,70]. Furthermore, low frequencies of circulating PMN-MDSCs and M-MDSCs are associated with higher overall survival in non-small cell lung cancer (NSCLC) patients treated with anti-PD-1 immunotherapy [71]. Accordingly, accumulation of a specific subset of circulating Tie2^hi^ M-MDSCs that suppress antitumor T-cell response has been reported to correlate with a poor clinical outcome in NSCLC patients [72]. In patients with metastatic melanoma treated with ipilimumab, low frequencies of circulating M-MDSCs prior treatment were a predictor of better clinical outcome [73]. Thus, circulating levels of MDSCs can be used as a predictive marker for immune checkpoint blockade-based therapies in different tumors [68].

MDSCs contribute to tumor progression through different mechanisms, including the induction of angiogenesis and EMT, the secretion of matrix metallopeptidase 9 (MMP9), VEGF (in STAT3-dependent manner), transforming growth factor (TGF)-β, and growth factors (i.e., EGF), and the promotion of pre-metastatic niches and immune evasion (Figure 3) [62,74]. Several studies suggest that MDSCs are recruited in the pre-metastatic niche, through the CXCL1–, CXCL2–, and CXCL5–CXCR2 axis and in response to the pro-inflammatory proteins S100A8 and S100A9. Once in the site, MDSCs stimulate the migration of tumor cells by secreting TNFα, CXCL2, TGFβ, IL-6 and CCL2 [75]. MDSCs also enhance cancer stemness, since in vitro co-culture of MDSCs with primary ovarian cancer cells increased cancer cell stemness and promoted tumor sphere formation, increasing the incidence of tumor and metastatic foci in a xenograft model [54].

The main feature of MDSCs is their strong immunosuppressive activity [76]. The mechanisms of MDSC-induced immunosuppression include the production of reactive oxygen (ROS) and reactive nitrogen species (RNS) that cause T-cell hypo-responsiveness and apoptosis, affecting T-cell fitness by downregulating CD3ζ-chain expression and reducing cytokine secretion (Figure 3), as observed in pancreatic cancer and melanoma [62,76]. MDSCs-mediated T-cell hypo-responsiveness is also induced metabolically through deprivation from extracellular space of the amino acids arginine and cysteine, which are required for T-cell activation and proliferation, as well as through depletion of tryptophan by overexpression of indoleamine-pyrrole 2,3-dioxygenase (IDO) [76]. Moreover, MDSCs in TME produce large amounts of TGFβ and IL-10, which, respectively, exert anti-proliferative effects on T cells, inhibiting IL-2 secretion, and promote both the Treg differentiation and M2 polarization of TAMs [76,77].

At the cellular level, MDSCs derange immune checkpoint pathways and several studies have reported that high PD-L1 expression on blood MDSCs of cancer patients correlates with disease stage, acting as negative regulators of T and NK cell functions in TME [78]. Interestingly, M-MDSCs are reported to be more immunosuppressive compared to PMN-MDSCs, both in tumor-bearing mice and in cancer patients, while tumor-infiltrating M-MDSCs display a more potent suppressive activity than splenic M-MDSCs [79].

M-MDSCs predominantly suppress T-cell activation through the production of ARG1, iNOS and TGFβ. Moreover, PMN-MDSCs are reported to primarily produce ROS and to exert antigen-specific immunosuppressive activities by cell–cell contact with T lymphocytes [76]. In addition, M-MDSCs exhibit higher cell plasticity compared to PMN-MDSCs, which is strictly controlled by specific transcription factors, such as c/EBPβ, nuclear factor κB (NF-κB) and STAT-3 [7]. Moreover, while in the TME M-MDSCs can differentiate into immunosuppressive TAMs [80], PMN-MDSCs are the predominant population in most cancers [81].

Although today we have a better understanding of both the immunosuppressive functions and metabolic traits of MDSCs, it remains to be established whether PMN-MDSCs and M-MDSCs can be subdivided into smaller and distinguishable subsets. In fact, due to the shared expression of common surface markers, the distinction of MDSCs from monocytes and neutrophils is still a great challenge for the design of effective MDSCs-targeted therapies.

PMN-MDSCs share the CD11b^+^CD14^−^CD15^+^ phenotype with mature neutrophils and currently can be separated from peripheral blood only by the density gradient. Recent data obtained by RNA-sequencing and single-cell approaches have indicated several potential markers of MDSCs in cancer settings; however, their clinical utility and reliability need to be established. Condamine et al. demonstrated in patients with NSCLC and head and neck cancer that PMN-MDSCs have a distinct transcriptional profile characterized by the enrichment of genes involved in ER stress response, M-CSF, IL-6, IFNγ and NF-κB, compared to neutrophils isolated from the same patients and from healthy donors. Moreover, this study identified lectin-type oxidized LDL receptor 1 (LOX1) as a specific surface marker of PMN-MDSCs in cancer patients [82]. In PDAC, transcriptomic analysis showed a distinct gene signature of M-MDSCs compared to monocytes, indicating STAT3 as a key regulator of monocytes reprogramming into M-MDSCs [79].

In a recent study, single-cell RNA-sequencing confirmed that both PMN-MDSCs and M-MDSCs isolated from spleens and tumors from breast cancer-bearing mice have a unique gene signature that differs from that of neutrophils and monocytes, though the expression of IL1β, ARG2, CD84 and WFDC17 identified an overlapping signature common to PMN-MDSCs and M-MDSCs [83]. Using single-cell analysis to compare tumor and normal tissue from early-stage NSCLC patients, Song et al. demonstrated the accumulation in tumor tissues of M-MDSCs expressing IL-10, CD14 and VEGF-A and PMN-MDSCs expressing IL-6, LOX1 and TGFβ1 [84].

### 4.3. Tumor-Associated Neutrophils (TANs)

Neutrophils are short-lived, terminally differentiated and non-proliferative myeloid cells involved in proliferation and dissemination of tumor cells, as well as in immune suppression [13]. TANs are present in the TME of many solid tumors, and a large body of evidence has proved their prognostic significance in both peripheral blood and tumor tissues of cancer patients [85]. A higher percentage of TANs are generally associated with poorer response to chemotherapy and radiotherapy in several cancers, except for ovarian and gastric cancers [13]. Moreover, the neutrophil-to-lymphocyte ratio (NLR) in the blood is a prognostic survival marker in different tumors [86]. However, the exact correlation between NLR and/or TANs frequency with clinical outcome remain elusive and can be attributed, at least in part, to the functional plasticity of TANs. Indeed, TANs have been described to exhibit both anti- and pro-tumor features [87]; yet the full spectrum of transcriptional states of TANs, particularly in patients, remains unknown.

A recent time-of-flight mass spectrometry (CyTOF) analysis has identified seven mature neutrophil subsets in the blood of melanoma patients [88]. In a mouse model of lung adenocarcinoma, a specific subset of TANs characterized by high expression of the sialic acid binding Ig-like lectin F (SiglecF^high^) and associated with several pro-tumor functions was described to accumulate in TME [89].

Single-cell RNA sequencing of TANs from human and mouse lung tumors revealed five and six neutrophil subsets, respectively, with particular subsets of TANs expressing canonical neutrophil markers (i.e., MMP8, MMP9, S100A8, S100A9, ADAM8). Of relevance, TANs’ subsets expressing inflammatory cytokines (i.e., CCL3, M-CSF) and expressing type I interferon-response genes are conserved between humans and mice [90,91].

The N1-N2 nomenclature has been used to distinguish neutrophil subpopulations with pro- vs. anti-tumor activity, respectively. N1 and N2 TANs are mainly defined based on their functional phenotypes; as specific cell surface markers have not yet been identified and apparently, they can mutually convert into each other [92]. N1 neutrophils are highly cytotoxic cells, display a more mature phenotype and high immune-activating ability. Conversely, N2 neutrophils are poorly cytotoxic, showing an immature phenotype and a high pro-angiogenic, pro-metastatic and immunosuppressive activity [86]. Recruitment of circulating neutrophils in tumor tissues is mainly regulated by CXCL1, CXCL2, CXCL8 and CXCL5 chemokines, the complement component anaphylatoxin C5a and tumor-derived oxysterols [93]. Tumor-derived factors dictate the phenotype and function of TANs. In particular, TGFβ has been demonstrated to induce a pro-tumor state characterized by high expression of arginase and strong immunosuppressive activity; on the other hand, IFNβ, IFNγ and GM-CSF stimulate TANs’ polarization into an anti-tumor phenotype characterized by high cytotoxic activity [92,94].

TANs themselves can influence the TME landscape, both directly and indirectly. In both murine and human tumors, TANs support the proliferation and extravasation of tumor cells and angiogenesis, and hijack antitumor immunity [13]. Angiogenic TANs produce a high level of pro-angiogenic factors and matrix metalloproteinases (i.e., VEGF, MMP9) [86]. MMPs favor angiogenesis through remodeling the extracellular matrix, as well as cancer cell migration and invasion by stabilizing integrins [95]. In addition, TANs dampen T-cell-mediated antitumor immunity and support immune evasion through the production of the immunosuppressive cytokine TGFβ, the upregulation of PD-L1, ROS production and the release of ARG1 [96,97].

Another important tumor-promoting mechanism is NETosis, a process by which neutrophils expel net-like structures (NETs) into the extracellular space. Cancer-primed neutrophils release NET during formation of spontaneous metastasis [98,99]. Mechanistically, during NETosis, neutrophils secrete the high mobility group box 1 (HMGB1) protein, thereby activating TLR9 signaling pathways, which promote cancer cells adhesion, proliferation and migration [100]. Moreover, in preclinical lung cancer, NET formation was reported to awake dormant cancer cells [101]. NETs can also act as a protective hull on cancer cells against cytotoxic immune T cell-mediated responses [102].

In contrast with these findings, TANs can also exert anti-tumor activities. They can produce high levels of ROS, NO and TNFα and express TNF-related apoptosis-inducing ligand (TRAIL), thus suppressing tumor cells proliferation. In response to the N1-polarizing cytokines IFNγ and GM-CSF, TANs acquire antigen-presenting cell (APC) features and the ability to stimulate T-cell proliferation [13,86].

## 5. Pre-Clinical Targeting of Myeloid Cells in Cancer

**Figure 4:**
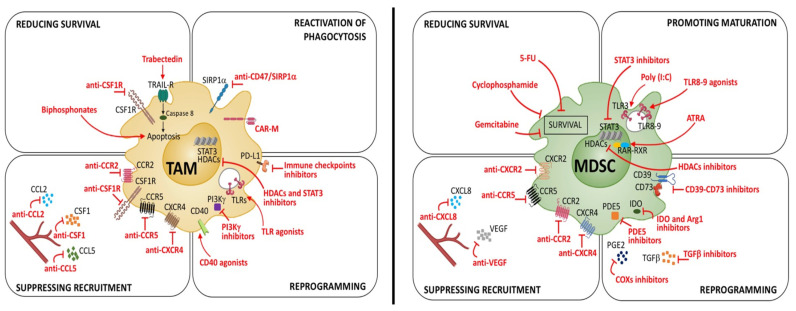
Main therapeutic approaches targeting TAM (left) or MDSC (right). Strategies targeting TAMs fall into four main categories: (1) direct killing of TAMs; (2) reactivation of their phagocytic activity; (3) inhibition of the recruitment of TAMs; and (4) re-education of TAMs towards a tumoricidal and immunostimulating phenotype. Similar to TAM, MDSC targeting can also be achieved through multiple approaches, including: (1) reducing their survival; (2) favoring their differentiation towards a mature myeloid effector phenotype; (3) inhibition of their recruitment, in both tumor tissues and secondary lymphoid organs; and (4) inhibition of their immunosuppressive functions. CSF1, colony stimulating factor 1; CSF1R, CSF1 receptor; PI3Kγ, phosphoinositide 3-kinase-γ; HDAC, histone deacetylase; RARs-RXRs, retinoid-activated transcriptional regulators; ATRA, all trans retinoic acid; PDE5, phosphodiesterase; 5-FU, fluorouracil. For abbreviations and details, see the text.

The growing understanding of the mechanisms underpinning the pro-tumoral activities of myeloid cells has paved the way for the development of multiple approaches to deplete or reprogram these cells in anti-tumor effectors [8]. Many of these preclinical approaches have been shown to exert significant anticancer effects and are now being evaluated in clinical trials (Figure 4).

### 5.1. TAMs Targeting Approaches

The neutralization of CSF1/CSF1R signaling by multiple approaches such as CSF1R-blocking antibodies and small molecules tyrosine kinase inhibitors has been demonstrated to efficiently deplete TAMs in several pre-clinical models such as fibrosarcoma, mesothelioma, colorectal, lung, prostate and pancreatic cancer [103]. Inhibiting the accumulation of TAMs by antibody-mediated CSF1R blockade was paralleled by both increased infiltration of CD4^+^ and CD8^+^ T cells and tumor growth inhibition [103]. Blocking CSF1R signaling can also reprogram macrophages in M1 effectors [104].

CCL2/CCR2 is a well-recognized axis driving the recruitment of both TAMs and MDSCs. Accordingly, CCR2 inhibitors reduce the infiltration of immunosuppressive myeloid cells limiting both primary tumor growth and metastasis spreading [105,106]. The anti-tumor effect was dependent on CD8^+^ T cells, supporting the concept that the depletion of tumor-associated myeloid cells mitigates immunosuppression and enhances the efficacy of T-cell targeting immunotherapies. The recruitment of myeloid cells is one of the pro-tumoral activities exercised by IL-1β, whose neutralization by an anti-IL-1 receptor (IL-1R) antibody curbed myeloid cell accumulation and tumor progression in mouse models of lung, breast and pancreatic tumors [107].

The marine-derived chemotherapeutic trabectedin, in addition of targeting tumor cells, selectively eliminates TAMs by activating caspase 8-dependent apoptosis through TRAIL [108]. An alternative strategy for TAMs’ depletion exploits their high expression of scavenging receptors (e.g., CD163, MRC1/CD206, MARCO and STAB1) to selectively deliver cytotoxic drugs into TAMs. For example, in a mouse model of melanoma, a CD163 antibody conjugated with a lipid carrier loaded with doxorubicin selectively eliminated CD163^+^ TAMs, enabling tumor regression [109].

Thanks to the inherent plasticity of macrophages, many studies have developed approaches aimed at reprogramming TAMs in immunostimulatory and tumoricidal cells.

Agonist stimulation of macrophage activating receptors, including TLRs and the TNF receptor family member CD40, can trigger anti-tumor immune responses. For example, the injection of TLR7/TLR8 agonists in a subcutaneous model of melanoma rewired macrophages into proinflammatory cells increased T-cell infiltration. These anti-tumor effects were further enhanced by the combination with checkpoint inhibitors (anti-CTLA4 and anti-PD-1 antibodies) [110]. CD40 agonists are reported to induce cytotoxic activity by TAMs in different tumor models, including pancreatic cancer [111]. Noteworthily, the combination of anti-CSF1R antibody with the agonist anti-CD40 antibody led to a synergistic induction of proinflammatory macrophage polarization and the activation of CD8^+^ T cells, resulting in the regression of several transplanted tumor models (e.g., colon, sarcoma, and breast cancer) [112].

Phosphoinositide 3-kinase γ (PI3Kγ), the most highly expressed PI3K isoform in myeloid cells is activated by many chemoattractant receptors and is associated with the recruitment of myeloid cells in murine and human tumors [113]. Moreover, in several tumor models, such as melanoma, lung, breast, head and neck carcinoma, genetic depletion or pharmacological inhibition of PI3Kγ-induced proinflammatory gene expression in TAMs triggered the infiltration and activation of CD8^+^ T cells, which in turn reduced tumor growth and metastases [114,115].

Various evidence indicates that the activation of complement cascade supports tumor-promoting inflammation rather than anti-tumor immunity. In a squamous cell carcinoma model, TAMs promoted C5a production and the consequent C5aR-mediated M2-polarization, resulting in CD8^+^ T-cell inhibition and cancer progression [116]. According to this, blocking C5aR signaling by the small molecule antagonist PMX-53 reprogrammed TAMs toward an M1 phenotype, enhancing the antitumor efficacy of PD-1/PD-L1 blockade [117]. Furthermore, in both transplanted and chemically induced sarcoma, genetic ablation of C3 and C3aR was associated with reduced accumulation and pro-tumoral skewing of TAMs, along with increased T-cell activation and response to anti-PD-1 therapy [118].

A growing number of studies are showing that metabolic changes are associated with different activation states of macrophages [119]. Enhanced aerobic glycolysis and pentose phosphate pathway (PPP), along with a break in the Krebs cycle (TCA cycle) characterizing M1 macrophages, are causally linked to the expression of inflammatory genes. In contrast, M2 macrophages fuel their energy needs via oxidative phosphorylation (OXPHOS) and β-oxidation of fatty acids (FAO), generating high levels of ATP and acetyl-CoA that participates in TCA cycle and cholesterol biosynthesis [120]. Although these are oversimplified models, they support the hypothesis that reprogramming of selected metabolic traits could be a useful strategy to enhance TAMs’ effector functions.

In lung and breast cancer models, TAMs showed an increased expression of key glycolytic enzymes (e.g., HK2, PFK, PKM2, and enolase1), suggesting that an accelerated glycolysis might be linked to the expression of pro-tumor activities [121]. Accordingly, dampening the glycolytic influx in TAMs by dichloroacetic acid significantly limited their migration and pro-metastatic ability [121]. Blocking glycolysis activity via HK2 inhibition also suppressed the vascular network formation and extravasation of tumor cells [122].

In multiple murine and human tumors, TAMs showed higher expression of the scavenger receptor CD36, associated with an increased uptake and breakdown of triglycerides by FAO. This metabolic commitment sustains the activation of STAT6, acting as a master coordinator of M2 gene expression [120]. Hence, targeting CD36 or FAO in macrophages might represent a potential strategy for their M1-reprogramming. Accordingly, in murine models of lymphoma and myeloma, either genetic ablation of CD36 in TAMs or FAO inhibition by etomoxir impaired macrophage pro-tumoral phenotype, hampering tumor growth and progression [120].

The production of α-ketoglutarate (αKG) via glutaminolysis is a key molecular checkpoint that promotes both oxidative metabolisms, by feeding the TCA cycle, and M2-gene expression by supporting Jmjd3-dependent H3K27 demethylation [123]. In line, glutamine production in TAMs was found to be associated with a pro-tumoral M2-like phenotype. Accordingly, limiting the glutamine pool through the ablation of glutamine synthase or the inhibition of glutamate–ammonia ligase (GLUL) switches M2-like TAMs toward the M1-like phenotype [123,124].

Epigenetic reprogramming is another attractive strategy to reshape gene expression and functional TAM activation. It is known that the efficacy of many epigenetic drugs currently used in the clinic depends on their direct effects on tumor cells, as well as on their ability to modulate anti-tumor immunity [125]. In a breast cancer model, the class IIA HDAC inhibitor TMP195 was able to reprogram TAMs in inflammatory anti-tumor cells and synergized with inhibition of PD-1 in reducing tumor burden and metastasis [126]. In human and murine mesothelioma models, inhibitors of the histone methyl transferase EZH2 have been found to be associated with the recruitment of monocytes that differentiate in pro-tumoral TAMs, which impair the cytotoxic activity of adoptively transferred M1 macrophages [127,128]. Nevertheless, in a prostate cancer model, EZH2 inhibition in tumor organoids was able to induce stimulator of interferon genes (STING)-dependent activation of genes involved in antigen presentation, Th1 chemokine signaling and interferon response, including PD-L1 gene expression [129]. In keeping with this epigenetic reprograming of cancer cells, EZH2 inhibition was found to be associated with M1 TAMs reprogramming, increased recruitment of activated CD8^+^ T cells and enhanced response to PD-1 blockade in vivo [129]. These studies indicate that targeting both histone acetylation and methylation might be exploited to reshape the TME composition, although the effects of epigenetic modulators might vary across different tumor types.

Blocking phagocytosis checkpoints is an additional promising strategy to foster the anti-tumor activities of macrophages [130]. Indeed, upregulation of “don’t eat me signals” by tumor cells is a well-recognized mechanism of immune evasion exploited by several cancers. The CD47–SIRPα axis is the most common “don’t eat me” axis, whose neutralization by anti-CD47 or anti-SIRPα antibodies can enhance phagocytic clearance of cancer cells in many preclinical tumor models [131]. Noteworthily, CD47 blockade in tumor cells can also enhance cross-presentation of tumor antigens for CD8^+^ T-cell activation, therefore improving anti-tumor effects [130]. Moreover, preclinical studies have demonstrated that neutralization of the CD47–SIRPα axis lowered the threshold for macrophage activation, enhancing the efficacy of various therapeutic antibodies such as rituximab in non-Hodgkin lymphoma, the anti-HER2 antibody in breast cancer, the anti-CD271 antibody in melanoma, and the anti-CD56 antibody in small-cell lung cancer [130]. Recently, the immune checkpoint inhibitor Hu5F9-G4, blocking CD47, was shown to synergize with rituximab to eliminate B-cell non-Hodgkin’s lymphoma cells by enhancing macrophage-mediated antibody-dependent cellular phagocytosis [132].

In addition to inhibiting PD1^+^ T effector cells, the expression of PD-L1 by cancer cells enables their evasion from macrophage-mediated phagocytosis. Although the mechanisms controlling expression and anti-phagocytosis function of PD-1 in TAMs are still unclear, the anti-tumor effect of blocking PD-1–PD-L1 axis in TAMs was definitely proved in mice lacking T, B and NK cells [133]. However, in transplant tumor models of melanoma, fibrosarcoma and colon cancer, genetic ablation of PD-1 in myeloid cells was associated with a remarkable anti-tumor effect, by favoring differentiation and functions of effector memory T cells [134]. Therefore, both phagocytosis-mediated and T cell-mediated anti-tumor immunity contribute to the therapeutic efficacy of PD-1–PD-L1 neutralization. In an attempt to enhance the phagocytosis capacity of macrophages, a CD47/PD-L1 bispecific antibody was developed and evaluated in mouse models, where it demonstrated higher efficacy than single anti-CD47 or anti-PDL1 treatment, both as monotherapy and in combinational therapy [135].

The leukocyte immunoglobulin-like receptor 1 (LILRB1) emerged as a phagocytosis inhibitory checkpoint that binds the β2-microglobulin (β2M) subunit of the histocompatibility complex class I (MHC-I). Preclinical studies pointed out that LILRB1 is highly expressed by TAMs and is responsible for the resistance of cancer cells expressing the common MHC-I component β2M to anti-CD47-induced phagocytosis [136]. Therefore, MHC class I–LILRB1 signaling axis, in addition to inhibit NK cells [137], provides an inhibitory axis whose neutralization could be exploited as anti-cancer approach.

Taking inspiration from chimeric antigen receptor (CAR)-T cells, endowing human macrophages with CAR represents an attractive strategy to overcome the inability of T cells to penetrate solid tumors. Macrophages genetically engineered to express a CAR specific to a tumor antigen are supposed to efficiently infiltrate solid tumors, where they could exert anti-tumor activity. Accordingly, primary human macrophages expressing a HER2-CAR have been generated and tested in vitro and in vivo in various preclinical xenograft models, demonstrating therapeutic efficacy, through increased phagocytosis of tumor cells and the conversion of bystander M2 macrophages to M1 polarization [138].

### 5.2. MDSCs Targeting Approaches

Elimination of MDSCs to alleviate immunosuppression and enhance anti-tumor immunity can be achieved by multiple strategies, including blocking their production during “emergency hematopoiesis”, inhibiting their recruitment in both tumor tissues and secondary lymphoid organs and promoting their differentiation towards mature myeloid effector cells [62].

Blocking the CCL2/CCR2 axis was reported to be effective in reducing MDSCs and tumor growth in different preclinical models [105,139]. In addition, inhibitors of the CCR5 chemokine receptor have been shown to be effective in preventing MDSCs accumulation and immunosuppressive functions, both in mice [140] and in humans [141]. Moreover, genetic and pharmacological inactivation of CXCR2, which is the major chemotactic receptor for PMN-MDSCs and neutrophils recruitment into tumors, was demonstrated to be effective in reducing tumor-infiltrating PMN-MDSCs and improving the response to anti-PD-1 in different pre-clinical model, such as head and neck [142] and colon [143] cancer. In a colitis-associated cancer model, tadalafil, an inhibitor of phosphodiesterase-5 (PDE5), directly impaired MDSCs’ infiltration in colonic tissue, reducing tumor development [144].

Anticancer drugs such as gemcitabine, 5-fluorouracil, docetaxel, doxorubicin and paclitaxel can also deplete MDSCs, thus enhancing the effector functions of T and NK cells [145]. Inducing apoptosis via death receptor 5 (DR5) agonists is a more tailored approach that exploits the upregulation of this TRAIL receptor by MDSCs [146]. The activation of liver X receptor (LXR) is another interesting strategy capable of selectively inducing MDSC apoptosis, relieving immunosuppression and enhancing anti-tumor immunity [147].

In several tumor models, prostaglandins E2 (PGE2) emerged as a key molecule driving both MDSCs’ expansion and immunosuppressive activities [148]. Beyond several immunosuppressive molecules (e.g., IDO, IL-10, ARG1, VEGF and PD-L1), which are induced by PGE2 in MDSCs [149,150], we recently reported that tumor-derived PGE2 drives p50 NF-κB-dependent epigenetic reprogramming of M-MDSCs, diverting their response to IFNγ toward NO-mediated immunosuppression in preclinical models of fibrosarcoma and melanoma [7]. Although PGE2 synthesis can be efficiently blocked by cyclooxygenase 2 (COX2) inhibitors, their prolonged systemic use can lead to severe side effects, and thus alternative and safer approaches are needed. Blocking specific PGE2 receptors, such as EP1/EP2, may provide an alternative safer approach to boost specific anticancer immunity in patients [7]. Pharmacological inhibition of fatty acid transport protein 2 (FATP2) is an additional approach to impair PGE2 synthesis in PMN-MDSC, blocking their immunosuppressive activities and improving anti-CTLA-4 efficacy [151].

Activation of retinoic acid receptor through the all-trans retinoic acid (ATRA) represents an effective strategy to promote MDSCs’ differentiation towards mature DCs and/or macrophages [152,153]. Noteworthy, in preclinical models of breast cancer, the combination of ATRA with VEGFR2 inhibitors and conventional chemotherapy increased the efficacy of anti-angiogenic therapy in association with a significant reduction in tumor growth [154].

TLR7/8 agonists also provide a strategy to induce MDSCs differentiation in anti-tumor effector mode. In a mouse model of colon cancer, the administration of R848 oriented the phenotype of MDSCs towards M1-like macrophages and improved the antitumor effect of oxaliplatin [155].

The transcription factor STAT3 is a key transcription factor active in both TAMs and MDSCs, whose targeting can rescue anti-tumor immune responses [156]. The conjugation of STAT3 siRNA or STAT3 decoy to cytosine-phosphorothioate-guanine (CpG) has been developed to tail the delivery of STAT3 inhibitor to myeloid cells. CpG-STAT3 inhibitors allowed the targeting of TLR9 expressing PMN-MDSCs, leading to their reprogramming in inflammatory anti-tumor cells in different hematological and solid tumor models [157].

Shaping MDSCs’ metabolism represents another approach to myeloid cell reprogramming with the aim of obtaining anti-tumor functions. Both expansion and immunosuppressive activities of MDSCs are tightly associated with their metabolic commitment toward the CD36-mediated uptake of fatty acid and their subsequent oxidation. Accordingly, both CD36 deletion and pharmacological inhibition of FAO blocked the immunosuppressive functions of MDSCs, improving the efficacy of either immunotherapy or low-dose chemotherapy [120,158].

Finally, similar to TAMs, epigenetic modulators can influence MDSCs’ differentiation and activities. Whereas treatments with the enhancer of zeste homolog 2 (EZH2) inhibitor GSK126 promoted the expansion of MDSCs, impairing antitumor immunity [159], entinostat, a class I histone deacetylase (HDAC) inhibitor, impaired MDSCs’ immunosuppressive functions, improving the anti-tumor effects of anti-PD-1 antibodies [160].

## 6. Clinical Advances in Targeting Tumor-Associated Myeloid Cells

Tumors are dynamic and heterogeneous tissues that rely on the complex relationship and balance instated between cancer cells and infiltrating immune cells. While strategies that potentiate the activity of cytotoxic CD8^+^ T cells with immune checkpoint inhibitors (such as monoclonal antibodies (mAbs) against CTLA4, PD1 and PDL1) have shown efficacy in the treatment of cancers, such as melanoma and lung cancer, in most cases, cancer cells’ polyclonality and immunosuppressive microenvironment mean that only a small fraction of patients fully respond to immunotherapy [161]. Several studies demonstrated that TAMs and MDSCs massively infiltrate cancer tissues and contribute to tumorigenesis by promoting angiogenesis, invasion and metastasis formation, cancer cell stemness, immunosuppression and resistance to therapy [162], pointing to TAMs and MDSCs as attractive targets for cancer immunotherapy. Here, we reported the most advanced clinical interventions targeting either TAMs or MDSCs.

### 6.1. Clinical Trials Targeting TAMs

Pre-clinical observations on the tumor-promoting functions of M2-polarized TAMs are strongly supported by clinical evidence correlating the high frequency of infiltrating TAMs with poor overall survival (OS) in many cancers [8]. However, due to the functional plasticity of these cells, higher frequencies of TAMs have also been found to predict a good prognosis in colorectal cancer, ovarian carcinomas and follicular lymphoma, where an M1-like status of TAMs was observed [163,164]. In light of this dual facet of TAMs, different clinical approaches were suggested for their manipulation in cancer therapy. These latter converge into two main approaches: (1) abrogating TAM enrichment (Table 1) and (2) re-educating immunosuppressive M2-like TAMs into M1-like immunostimulatory and tumoricidal cells (Table 2). These two perspectives were approved for clinical trials by the Food and Drug Administration (FDA) agency. Here, we will focus on the most advanced strategies, as summarized in Table 1 and Table 2.

#### 6.1.1. Abrogating TAM Enrichment

As mentioned, a high frequency of TAMs in the tumor microenvironment is associated with both bad prognosis and immunosuppression. Therefore, therapeutic strategies to hamper their enrichment have targeted: (i) TAMs depletion and/or (ii) inhibition of their recruitment into TME (Table 1).

*Depleting TAMs*

A main approach to deplete TAMs is the inhibition of colony-stimulating factor 1 receptor (CSF-1R) which, interacting with its CSF-1 or IL-34 ligands, plays a critical role in the survival, differentiation and maturation of macrophages [165]. Several small molecule inhibitors or blocking antibodies were exploited to reduce the survival of macrophages. Pexidartinib (PLX3397) was approved in a phase 3 trial for the treatment of tenosynovial giant cell tumor (TGCT), which is characterized by high infiltration of CSF1R^+^ macrophages [166]. Pexidartinib is now under clinical evaluation for the treatment of breast, pancreatic and colorectal cancer in combination with other chemo- and/or immuno-therapies (see Table 1). Other CSF1R^+^ inhibitors include: the c-Fms inhibitor edicotinib (JNJ-40346527), in the treatment of prostate cancer (ClinicalTrials: NCT03177460); the kinase inhibitor vimseltinib, in the treatment of sarcomas, as well as TGCT (NCT04242238, NCT05059262). A number of monoclonal antibodies (mAbs) blocking the CSF1/CSF1R axis are under clinical development: cabiralizumab (FPA008) for the treatment of pancreatic, non-small cell lung and renal cell cancer, in addition to TCGT and melanoma (see Table 1); emactuzumab (RG7155) in combination with bevacizumab (anti-VEGF mAb) and paclitaxel is under evaluation for the treatment of ovarian cancer (NCT02923739); MCS110 mAb was instead approved for a phase 1/2 clinical trial for the treatment of melanoma in combination with BRAF/MEK inhibitors (NCT03455764). However, clinical phase 2 studies of pexidartinib in recurrent glioblastoma [167] and MCS-110 in triple negative breast cancer [168] indicated that these agents did not improve the outcome of patients, although therapies were well-tolerated. This suggests that patient stratification could be a necessary assessment in future studies.

*Inhibition of TAMs Recruitment*

A number of cytokines and chemokines regulate the trafficking of bone marrow-derived monocytes into the tumor microenvironment where they differentiate into TAMs. CCL2, which interacts with its receptor CCR2, has gained clinical relevance. In several cancers, CCL2 levels correlate with TAM frequency, metastasis score and poor survival [169].

At present, two main drugs that target CCR2 are under clinical evaluation: the CCL2-blocking monoclonal antibody carlumab (CNTO-888) and the small molecule CCR2-inhibitor PF-04136309. Carlumab showed a partial reduction in CCL2 levels, with good tolerance in patients affected by different solid tumors, while it did not show significant efficacy in a phase II study on castration-resistant prostate cancer patients [170]. In advanced pancreatic cancer patients, the PF-04136309 inhibitor in combination with FOLFIRINOX exerted an objective anti-tumor effect, as compared with FOLFIRINOX alone [171]. Recently, a tolerability study of CCR2-blocking antibody plozalizumab (MLN1202) was performed on melanoma patients (NCT02723006).

CCL5/CCR5 is another important axis for the recruitment of TAMs into TME [172]. Different CCR5 antagonists, formerly developed for the treatment of HIV, are under clinical studies for cancer therapy. These include: leronlimab (PRO 140), which is currently in a phase 1 study, either in combination with carboplatin or alone, for the treatment of triple-negative breast cancer [172], and in a phase 2 study for the treatment of solid metastatic tumors (NCT0450494); maraviroc and vicriviroc, plus pembrolizumab, were used in a phase 1 clinical trial in the treatment of metastatic colorectal cancer with a good toxicity profile (NCT03274804, NCT03631407). Furthermore, BMS-813160, a CCR2/CCR5 dual antagonist, has been studied in combination treatments in non-small cell lung cancer (NSCLC), hepatocellular carcinoma (HCC), and pancreatic ductal adenocarcinoma (NCT04123379, NCT03496662).

The CXCL12 chemokine and its receptor, CXCR4, represent another important gate for the mobilization and recruitment of monocyte/macrophage into TME [173]. Increased CXCR4 expression was associated with disease progression of NSCLC, while CXCL12 was increased after radiotherapy in different tumors [174,175]. Plerixafor (AMD3100), a CXCR4 antagonist, was used in combination with chemo-radiotherapy for the treatment of glioblastoma and studied for its ability to prevent the recurrence of glioblastoma after radiation treatment (NCT03746080). Another CXCR4 antagonist, motixafortide (BL-8040), combined with pembrolizumab in metastatic pancreatic cancer, is being evaluated (NCT02907099).

#### 6.1.2. Re-Education of TAMs

Although direct depletion of TAMs was shown to have effective antitumor functions, the heterogeneity of TAMs and in particular the antitumor potency of M1-like TAMs appears as a promising therapeutic option (Table 2).

*Targeting TAM Polarization*

TLRs are pattern recognition receptors that potently activate innate immune responses, favoring the pro-inflammatory polarization of macrophages. Therefore, several TLR agonists are under extended clinical evaluation. Of relevance, bacilli calmette guerin (BCG) is the first FDA-approved TLR agonist for the treatment of high-grade nonmuscle-invasive bladder cancer. It is capable to stimulate TLR2 and TLR4, promoting a conversion of TAMs toward an M1-like status [176]. Imiquimod, a TLR7 agonist, showed a partial response associated with changes in the inflammatory profile in breast cancer patients with skin metastasis [177]. 852A is another TLR7 agonist which has been tested for the treatment of melanoma and gynecological cancers [178]. The TLR8 agonist motolimod (VTX-2337), in combination with cetuximab, showed a significant benefit in human papilloma virus (HPV)-positive head and neck cancer patients [179]. IMO-2055 (TLR9 agonist) were evaluated in the treatment of colorectal cancer (CRC) and NSCLC patients, in combination with standard therapies showing a potential antitumoral effects, as well as a good tolerability (NCT00719199, NCT00633529, [180]). While resiquimod, a TLR7/8 agonist, showed an immunomodulatory effect on melanoma patients (NCT00960752), another TLR9 agonist, tilsotolimod, was tested in combination with standard immune checkpoint inhibitors (ICIs) in the treatment of advanced melanoma patients, showing beneficial effects as compared with ICIs alone (NCT03445533).

CD40 belongs to the TNF receptor superfamily and is expressed by APCs, including macrophages. The CD40 ligand (CD40L) is mainly expressed by T cells. The CD40–CD40L interaction upregulates the expression of MHC molecules and the production of pro-inflammatory cytokines, such as IL-12, both prototypical markers of M1-like macrophages [181]. Several anti-CD40 agonistic antibodies and CD40 ligands have been designed. Selicrelumab (RO7009789) and sotigalimab (APX005M) monoclonal antibodies are currently under clinical trials in the treatment of different solid tumors (e.g., pancreatic cancer, melanoma, sarcomas, pediatric neurological cancer) [181,182].

Interestingly, unlikely other Fc receptor agonists, the antibody Fc domain with inhibitory FcγRIIb is required for the anti-CD40 antibody because of its agonistic immunostimulatory activity. CP-870893, an IgG2 anti-CD40 antibody, was more effective in inducing immunostimulation [181]. CP-870893 showed anti-tumor activity in patients with different solid tumors (NCT00607048), as well as in pancreatic cancer and in mesothelioma patients [181]. Of note, ABBV-428 is a mesothelin-CD40 bispecific molecule currently studied in a phase 1 clinical trial in combination with nivolumab for the treatment of patients with advanced solid tumors (NCT02955251).

PI3Ks are involved in almost all types of intracellular signaling. The class 1b PI3Kγ is the only isoform expressed in myeloid cells and can inhibit NF-κB activation and, eventually, the pro-inflammatory phenotype of macrophages. Moreover, PI3Kγ signaling drives the L-arginine metabolism from iNOS enzymatic activity toward the ARG1-mediated degradation, a crucial pathway for immunosuppression [114]. Of relevance, low activity of PI3Kγ in head and neck lung cancer patients correlated with better prognosis and longer overall survival [114]. Eganelisib (IPI-549), a selective PI3Kγ inhibitor, is currently being tested in phase 1b clinical trials in combination with different standard therapies (e.g., doxorubicin, paclitaxel, nivolumab, bevacizumab), in triple-negative breast, non-small cell lung, head and neck, urothelial cancers and melanoma [183].

Histone deacetylases (HDACs) are responsible for removing the acetyl groups on histones, a crucial process in epigenetic regulation of gene expression. Tucidinostat (chidamide) inhibits Class I HDAC1, HDAC2, HDAC3, as well as Class IIb HDAC10, and has been approved by Chinese and Japanese FDA to be tested in clinical trials for the treatment of urothelial and gynecological cancers (NCT04562311, NCT04192903, NCT04651127).

*Re-Activation of Phagocytosis*

Myeloid cells, including macrophages, express SIRPα. Since CD47 is upregulated in both solid and hematological tumors and such overexpression is correlated with poor patient survival or poor response to therapy, several CD47-SIRPα antagonists were developed and are currently active in clinical trials [184]. These include: magrolimab (Hu5F9-G4), TTI-621, CC-95251, CC-90002 and STI-6643. Magrolimab is still under evaluation for the treatment of ovarian, breast, head and neck carcinomas, osteosarcoma, neuroblastoma, as well as hematological malignancies (see Table 2) [185]. TTI-621 is a fully human recombinant protein that blocks the CD47–SIRPα axis and improves the killing of cancer cells [185]. TTI-621 promoted macrophage-mediated tumor killing in a wide array of solid and hematologic malignancies. Currently, TTI-621 is also being tested on hematological neoplasms, leiomyosarcoma and multiple solid tumors (NCT02663518, NCT02890368, NCT04996004).

*Macrophage Engineering*

As mentioned above, genetic engineering approaches aimed to express chimeric antigen T cell receptor (CAR) against cancer-specific antigens has been developed [186]. More recently, academic laboratories and companies are developing CAR-expressing macrophages to selectively target tumor antigens. Notably, Klichinsky et al. described an anti-HER2 CAR-macrophage (CAR-M, CT-0508), endowed with antigen-specific phagocytic activity, significantly reduced metastatic tumor burden in humanized mouse cancer model [138]. Of relevance, based on impressive preclinical results, the US FDA recently approved a phase 1 clinical trial for the treatment of HER2^+^ cancers (NCT04660929) [187].

### 6.2. Clinical Trials Targeting MDSCs

Although the history of the identification and characterization of MDSCs is much more recent as compared to TAMs, the efforts made for their therapeutic targeting in cancer are increasingly providing promising results. Indeed, while the identity of MDSCs is rather challenging, a number of clinical trials are ongoing, pursuing strategies that reduce their frequency [188]. As MDSCs and TAMs are ontologically and functionally akin, several strategies for their targeting overlap. Indeed, some clinical trials are evaluating the effects of therapeutic agents on both TAM and MDSC populations (e.g., anti-CCR2 (NCT02345408), anti-CCR5 (NCT03184870), anti-CXCR4 (NCT04058145)). Table 3 describes the major ongoing clinical trials targeting MDSCs.

#### 6.2.1. Abrogating MDSCs Enrichment

*Inhibition of MDSCs Recruitment*

As with TAMs, the inhibition of MDSC trafficking to the tumor site is a promising strategy. The CXCL8 (IL-8) chemokine through its binding to CXCR1 or CXCR2 receptors supports tumor progression, partially promoting neutrophils and PMN-MDSCs recruitment [33]. In pre-clinical models, CXCR2 inhibition showed reduced MDSC frequency, increased T-cell infiltration, decreased tumor progression, as well as improved response to anti-PD-1 treatment [189].

At present, several CXCR1/2 inhibitors have been tested. SX-682, reparixin, navarixin and AZD5069 are the most studied, in combination with canonical chemotherapies, as well as with ICIs, showing promising results in terms of both tolerability and clinical outcome [190,191]. Recently, an anti-CXCL8 antibody, HuMax-IL-8, was confirmed to be safe and tolerable in patients with early-stage solid cancers and is currently under investigation in a phase 1/2 clinical study, in combination with nivolumab; however, indications about MDSCs’ frequency and responsiveness to therapy have not been provided yet (NCT03400332).

CXCL12 levels were associated with CXCR4^+^ MDSCs accumulation in patients with ovarian cancers [192]. Two CXCR4 inhibiting agents, plerixafor and motixafortide, are now under investigation for the treatment of head and neck and pancreatic carcinoma patients, in which the MDSCs will be monitored (NCT04058145, NCT03193190). Interestingly, VEGF is an indispensable stimulator of mobilization and expansion of MDSCs expressing the VEGF receptor 1 (VEGFR1) [193]. Several clinical studies with anti-VEGF/VEGFR therapies (bevacizumab) demonstrated inhibitory effects on MDSCs’ accumulation, in association with the inhibition of angiogenesis. Indeed, bevacizumab-based therapy significantly reduced the proportion of PMN-MDSCs in the peripheral blood of NSCLC patients [194]. Another study on patients with colorectal cancer showed that the FOLFOX regimen plus bevacizumab decreased PMN-MDSCs’ frequency, as well as providing a better clinical outcome [195].

*Depletion of MDSCs*

Low-dose chemotherapy has been shown to exert immunomodulatory effects by eliminating MDSCs and reducing their immunosuppressive capability [196]. Gemcitabine and fluorouracil (5-FU) are the two most studied cytotoxic agents for MDSCs depletion in cancer bearers [197]. Multiple studies on gemcitabine, fluorouracil, as well as capecitabine and cyclophosphamide, are under clinical evaluation or have already demonstrated efficacy in combination with immunotherapies (e.g., DC vaccine, ICIs), resulting in decreased MDSC numbers and benefiting the survival of cancer patients (see Table 3) [196,198]. However, other cytotoxic drugs such as cyclophosphamide can induce the opposite result, inducing MDSCs’ infiltration and expansion [199].

**Table 3:** Summarized list of completed or active clinical trials targeting MDSCs.

Strategy	Target	Drug Name	Combined Therapy	Disease	Clinical Trial
Inhibition of recruitment, mobilization, expansion	CXCR1/2-CXCL8	SX-682	Nivolumab	Metastatic Colorectal Cancer	NCT04599140
			Nivolumab	Pancreatic Cancer	NCT04477343
			Pembrolizumab	Metastatic Melanoma	NCT03161431
			BinTrafusp Alfa, CV301	Advanced Solid Cancer	NCT04574583
		Navarixin	Pembrolizumab	Advanced Solid Cancer	NCT03473925
		Reparixin	Paclitaxel	Metastatic Breast Cancer	NCT02370238
			Paclitaxel	HER2-neg Metastatic Breast Cancer	NCT02001974
			Single Agent	Early Breast Cancer	NCT01861054
	CXCR2	AZD5069	Enzalutamide	Metastatic Prostate Cancer	NCT03177187
			Nab-paclitaxel, Gemcitabine, MEDI4736	Metastatic Pancreatic Ductal Carcinoma	NCT02583477
			AZD9150, MEDI4736, Tremelimumab	Head and Neck Carcinoma	NCT02499328
	CXCR4	Plerixafor	Pembrolizumab	Head and Neck Cancer	NCT04058145
		Motixafortide	Atezolizumab	Metastatic Pancreatic Adenocarcinoma	NCT03193190
	VEGF/VEGFR	Bevacizumab	Capecitabine	Glioblastoma	NCT02669173
			Pazopanib Hydrochloride	Renal Cell Cancer	NCT01684397
			Anakinra	Metastatic Colorectal Cancer	NCT02090101
		Cabozantinib	Single agent	Prostate Cancer	NCT03964337
Depleting MDSCs	Whole cell	Gemcitabine	Nivolumab	Non-small Cell Lung Cancer	NCT04331626
			Modified vaccine expressing p53	Gynecological Cancers	NCT02275039
			DC vaccine	Breast Cancer	NCT02479230
			DC vaccine, imiquimod	Sarcomas	NCT01803152
		Fluorouracil	Avelumab, Cisplatin, Mitomycin	Bladder Cancer	NCT03617913
			Aldesleukin, Chemotherapies	Pancreatic Cancer	NCT02620865
		Capecitabine	Avelumab	Colorectal Cancer	NCT03854799
			Cisplatin, Rituximab	Head and Neck Squamous Cell Cancer	NCT04361409
		Cyclophosphamide	iNKT cells, hrIL-2	Hepatocellular Carcinoma	NCT04011033
			Pembrolizumab, Vit D, Aspirin	Gynecological Cancer	NCT03192059
Promoting MDSC differentiation	TLRs	Poly ICLC	IMA 950	CNS Tumor	NCT01920191
		Imiquimod	DC vaccine	Glioblastoma	NCT01808820
		Motolimod	Cetuximab, Nivolumab	Head and Neck Squamous Cell Cancer	NCT02124850
		CpG	Nivolumab	Pancreatic Cancer	NCT04612530
	RAR/RXR	ATRA	Ipilimumab	Melanoma	NCT02403778
			Pembrolizumab	Melanoma	NCT03200847
			Vaccine, Cyclophosphamide	Lung Cancer	NCT00601796
			Paclitaxel, p53-DC vaccines	Small Cell Lung Cancer	NCT00617409
	STAT3	Danvatirsen	Durvalumab	Pancreatic, Colorectal, Lung Cancer	NCT02983578
			Durvalumab	Non-Small Cell Lung Cancer	NCT03794544
Inhibiting suppressive functions	TGFβ	ABBV-151	Budigalimab	Advanced Solid Cancer	NCT03821935
		Pirfenidone	Atezolizumab	Advanced Non-Small Cell Lung Cancer	NCT04467723
		NIS793	PDR001	Advanced Solid Cancer	NCT02947165
		SAR439459	Cemiplimab	Advanced Solid Cancer	NCT04729725
		Bintrafusp alfa	Single agent	Advanced Solid Cancer	NCT02517398
			Single agent	Advanced Solid Cancer	NCT02699515
			Single agent	HPV-associated malignancies	NCT03427411
			Cheotherapy	Non-Small Cell Lung Cancer	NCT03840915
	COX2	Acetylsalicylic acid	Pembrolizumab, Clopidogrel	Head and Neck Cancer	NCT03245489
		Celecoxib	DC vaccine, cisplatin	Ovarian Cancer	NCT02432378
			Nivolumab, Ipilimumab	Colorectal Cancer	NCT03026140
			Glucoferon, Rintatolimod	Metastatic Breast Cancer	NCT03599453
	PDE5	Tadalafil	Single agent	Head and Neck Cancer	NCT01697800
			Anti-Tumor Mucin-1 Vaccine	Head and Neck Squamous Cell Cancer	NCT02544880
	HDACs	Entinostat	Ipilimumab, Nivolumab	Breast Cancer	NCT02453620
			Nivolumab	Pancreatic Cancer	NCT03250273
			Azacitidine, Nivolumab	Non-Small Cell Lung Cancer	NCT01928576
	NRF2	Omaveloxolone	Ipilimumab, Nivolumab	Melanoma	NCT02259231
			Single Agent	NSC Lung Cancer, Melanoma	NCT02029729
Modulation of MDSC metabolism	CD39/CD73	TTX-030	Pembrolizumab, Chemotherapies	Advanced Solid Cancer	NCT04306900
		SRF617	Chemotherapies, Pembrolizumab	Advanced Solid Cancer	NCT04336098
		Oleclumab	Durvalumab	Muscle Invasive Bladder Cancer	NCT03773666
			Durvalumab	Lung and Renal Cancer	NCT04262375
			Durvalumab	Head and Neck, Lung, Pancreatic Cancer	NCT04262388
			Paclitaxel, Carboplatin, MEDI4736	Triple Negative Breast Cancer	NCT03616886
			Durvalumab	Sarcomas	NCT04668300
	IDO	Indoximod	Docetaxel, Paclitaxel	Metastatic Breast Cancer	NCT01792050
		Epacadostat	Pembrolizumab	Melanoma	NCT02752074
		BMS-986205	Nivolumab, Radiation, Temozolomide	Glioblastoma	NCT04047706
	ARG1	INCB001158	Retifanlimab	Advanced Solid Cancer	NCT03910530
			Epacadostat, Pembrolizumab	Advanced Solid Cancer	NCT03361228
			Pembrolizumab	Advanced Solid Cancer	NCT02903914
			Chemotherapies	Advanced Solid Cancer	NCT03314935
	LXRs	RGX-104	ICIs, Chemotherapies	Advanced Solid Cancer, Lymphoma	NCT02922764

#### 6.2.2. Re-Education of MDSCs

*Promoting MDSCs Maturation*

The immature phenotype of MDSCs represents another promising target to reduce their accumulation and to overcome their immunosuppressive functions. In this regard, polyinosinic-polycytidylic acid (Poly ICLC), a synthetic double-stranded RNA ligand for TLR3 used as an immunostimulatory adjuvant, showed effects in reducing MDSCs frequency and related immunosuppression [200]. Currently, Poly ICLC is being evaluated for the treatment of central nervous system (CNS) tumors, and for its effect on MDSCs and Tregs frequency. However, while safety, tolerability and clinical outcomes showed positive results, MDSCs’ frequency and functions were not provided (NCT01920191) [201]. TLR7/8 agonists synergize with immunotherapeutic approaches to enhance antitumor efficacy, by preventing MDSCs suppressive functions [202,203]. In a phase 1 clinical trial (NCT02124850) in HNSCC patients, the TLR8 agonist motolimod in combination with cetuximab reduced the MDSCs’ frequency, inducing pro-inflammatory monocytic differentiation in tumor tissues [204]. CpG motifs, agonists of TLR9, have antitumoral immune activity as therapeutic vaccine adjuvants [205]. In addition, a clinical study in pancreatic cancer patients is testing the combination of CpG with nivolumab for safety and efficacy, evaluating the effects elicited on MDSCs (NCT04612530).

ATRA is a derivative of vitamin A with agonist activity towards retinoid-activated transcriptional regulators (RARs and RXRs). ATRA induces the maturation of immature myeloid cells into fully differentiated and less immunosuppressive cells [206]. ATRA was approved by the FDA as a standard treatment for acute promyelocytic leukemia (APL), as it promotes terminal differentiation of immature myelocytic tumor cells [207], and consequently proposed for the differentiation of immature MDSCs into macrophages and DCs [208]. A clinical trial in renal cell carcinoma (RCC) demonstrated that ATRA treatment reduced total CD33^+^ MDSCs, and induced a stable disease in the majority of patients [209]. Another trial on metastatic melanoma patients tested ATRA in combination with standard ipilimumab therapy, proving a reduced number of circulating MDSCs as compared with ipilimumab therapy alone (NCT02403778) [210]. Furthermore, the combination of ATRA with a p53-transduced DC vaccine in SCLC patients showed a reduced number of total and M-MDSCs and improved the anti-cancer immune response. However, no clinical outcomes have been reported from this trial [153]. STAT3 activation is a key event regulating expansion and immunosuppressive functions of MDSCs, preventing their terminal differentiation [79]. Among others (see Table 3), a phase 1 trial (NCT01563302) revealed that systemic administration of danvartisen, an antisense oligonucleotide inhibitor of STAT3, reduced the levels of peripheral PMN-MDSCs in patients with diffuse large B-cell lymphoma (DLBCL) [211]. Moreover, a phase II clinical trial tested the AZD9150 STAT3 inhibitor in combination with ICIs in solid tumor patients (NCT02499328).

*Inhibition of MDSCs Immunosuppressive Functions*

TGFβ mediates several immunosuppressive activities during tumor development, including expansion and induction of immunosuppressive MDSCs [76]. Accordingly, several strategies targeting TGFβ are under clinical evaluation. These include TGFβ inhibitors (ABBV-151, pirfenidone) and blocking antibodies (NIS793, SAR439459) (Table 3). Interestingly, bintrafusp alpha (M7824), a bispecific fusion protein blocking both PD-L1 and TGFβ, is under clinical evaluation for the treatment of different solid tumors, where the frequency of immunosuppressive MDSCs is being characterized (Table 3).

PGE2 is involved in inflammation, angiogenesis, tumor progression via MDSCs recruitment, ARG1 upregulation and regulation of PD-L1 expression on tumor-infiltrating MDSCs [212,213], promotion of CXCL12/CXCR4-mediated recruitment of MDSCs [192]. PGE2 is synthesized from arachidonic acid by cyclooxygenases (COXs). Celecoxib, a selective inhibitor of COX-2, has been of great interest as a treatment suppressing MDSC functions, alone and in combination with ICIs. Various clinical trials combining Celecoxib with standard therapies are currently ongoing, characterizing MDSCs’ enrichment and functions [214]. However, COX inhibitors showed adverse effects as a result of pan-inhibition of prostanoid production; therefore, targeting the downstream receptors of PGE2 (e.g., prostaglandin E receptors/EPs) can be a more beneficial approach [215]. In a phase I clinical trial in patients with advanced solid tumors, an EP4 inhibitor significantly enhanced tumor infiltration of CD3^+^ and CD8^+^ T cells, while the levels of MDSCs in these patients were not reported (NCT02540291).

Phosphodiesterase 5 (PDE5) inhibitors, such as tadalafil, have been reported to downregulate the expression of ARG1, iNOS, and IL-4Ra in MDSCs [216]. Tadalafil treatment in metastatic melanoma and HNSCC patients proved to be safe and able to significantly reduce MDSCs accumulation, as well as ARG1 and iNOS activity [217,218]. Moreover, a phase I trial testing tadalafil and a telomerase vaccine (GV1001), alongside gemcitabine, is ongoing in patients with locally advanced pancreatic adenocarcinoma (NCT01342224).

As with TAMs, HDAC inhibitors induced a significant reduction in ARG1 and COX-2 expression in MDSCs, impaired MDSC trafficking and promoted their differentiation towards a macrophage-like phenotype, improving the response to immunotherapeutic agents [219,220]. The class I HDAC inhibitor, entinostat, is currently under study in different clinical trials. While two clinical trials on breast and ovarian cancer (NCT02708680, NCT02915523) failed to improve the clinical response, other studies combining entinostat with ICIs are underway for the treatment of breast, pancreatic and non-small cell lung cancer (NSCLC) [221] (Table 3).

*Modulation of MDSCs Metabolism*

The ectonucleotidases CD39 and CD73 catalyze the conversion of ATP/ADP to adenosine, which play a pivotal role in immunosuppression. Significant expression of CD39/CD73 was detected on the surface of MDSCs in lung and colon cancer patients, and was significantly associated with the response to chemotherapy, and hence was suggested to promote angiogenic process [222]. Therefore, many strategies inhibiting CD39/CD73 have been explored in clinical trials, in combination with ICIs. Among these inhibitors, TTX-030, SRF617 and oleclumab (MEDI9447) are being tested in bladder, lung, renal, breast cancers (Table 3). Tryptophan catabolism via the activity of IDO enzyme is a generally accepted mediator of immunosuppression in tumors and IDO expression is positively correlated with disease stage in many human cancers [223]. IDO is highly expressed by tumor-infiltrating immune cells, such as MDSCs [224]. Although the inhibition of IDO with epacadostat in combination with pembrolizumab failed in improving melanoma patients outcome [225], other phase III studies on pembrolizumab plus epacadostat showed a higher response rate in different solid tumors, as compared to control groups (NCT03361865; NCT03374488; NCT03260894; NCT03358472). Moreover, other IDO inhibitors, such as indoximod, in combination with the prostate cancer vaccine sipuleucel-T showed a positive clinical response (NCT01560923). Other drugs which regulate MDSCs include ARG1 inhibitor (INCB001158) (Table 3) [226], metformin [227], LXRs agonist RGX-104 (NCT02922764), and vitamin D3 [228].

## 7. Conclusions and Future Perspective

Although specific immunity is rightly considered the effector arm of antitumor response, and numerous strategies have been devised to reinforce specific lymphocyte responses in cancer patients, it is increasingly evident that the expansion of myeloid populations induced by growing tumors dramatically interferes with specific antitumor immunity and with the efficacy of anticancer therapies. Furthermore, new antitumor strategies (e.g., CD47/SIRPα antagonists) are being defined, aimed at the reactivation of cytotoxic properties typical of innate immunity. Therefore, the future integration of strategies involving both innate and specific immunity seems no longer postponable, as well as on the basis of new knowledge that points to myeloid cells as a powerful protumoral immune checkpoint. The mechanisms that drive “emergency myelopoiesis” in cancer patients and the functional integration of the multistep events leading to the development of the suppressor phenotype of myeloid cells are therefore to be considered as essential biological traits of tumor progression. Future studies will therefore have to better understand the functional integration of the processes that contribute to establishing protumoral myeloid conditions, both at a systemic and intratumor level. This may lead to the optimization of strategies aimed at the functional misalignment of what appears to be an interconnected multistep process of protumoral reprogramming. This multitargeting approach will probably make tumors more attackable from a therapeutic point of view, restoring effective cooperation between innate and specific antitumor responses.

Achieving this goal will require the acquisition of new basic knowledge and its translation into new clinical studies evaluating the effects of drugs targeting the immunosuppressive myeloid compartment in combination with standard therapies and/or immunotherapies.

## Figures and Tables

**Table 1:** Summarized list of completed or active clinical trials targeting TAMs enrichment.

Strategy	Target	Drug Name	Combined Therapy	Disease	Clinical Trial	Clinical Benefit Rate
TAM depletion	CSF1/CSF1R	Pexidartinib	Single agent	Tenosynovial Giant Cell Tumor (TGCT)	NCT02371369	Overall response: 53%
			Paclitaxel	Advanced solid tumors	NCT01525602	Clinical benefit: 40%
			Single agent	Acute Myeloid Leukemia	NCT01349049	Overall response: 21%
			Durvalumab	Advanced Pancreatic and Colorectal Cancer	NCT02777710	No results posted
			Eribulin	Metastatic Breast Cancer	NCT01596751	Not yet reported
		PLX7486	Single agent	Advanced solid tumors, TGCT	NCT01804530	No results posted
		BLZ945	Spartalizumab	Advanced solid tumors	NCT02829723	Not yet reported
		Edicotinib	Daratumumab	Advanced Prostate Cancer	NCT03177460	Not yet reported
		ARRY-382	Pembrolizumab	Advanced Solid Tumors	NCT02880371	No results posted
		IMC-CS4	GVAX, Pembrolizumab	Pancreatic Cancer	NCT03153410	Not yet reported
			Durvalumab, Tremelimumab	Advanced Solid Tumors	NCT02718911	Disease Control: 33.3%
			Vemurafenib, Cobimetinib	Melanoma	NCT03101254	Not yet reported
		Emactuzumab	Atezolizumab	Advanced Solid Tumors	NCT02323191	No results posted
			Paclitaxel	Advanced Solid Tumors	NCT01494688	Overall Response: 71%
			Bevacizumab, Paclitaxel	Ovarian, Fallopian Tube or Peritoneal Cancer	NCT02923739	Not yet reported
		Cabiralizumab	Single agent	Tenosynovial Giant Cell Tumor	NCT02471716	Not yet reported
			Nivolumab	Advanced Solid Tumors	NCT02526017	Not yet reported
			Nivolumab, chemotherapies	Advanced Pancreatic Cancer	NCT03336216	Not yet reported
			Sotigalimab, Nivolumab	Melanoma, NSC Lung, Renal Cell Carcinoma	NCT03502330	Not yet reported
		Vimseltinib	Avelumab	Advanced or Metastatic Sarcomas	NCT04242238	Not yet reported
			Single agent	Tenosynovial Giant Cell Tumor	NCT05059262	Not yet reported
		AMG 820	Pembrolizumab	Advanced Solid Tumor Cancer	NCT02713529	Overall Response: 34%
		Axatilimab	Durvalumab	Solid Tumors	NCT03238027	Not yet reported
			Durvalumab	Unresectable Cholangiocarcinoma	NCT04301778	Not yet reported
		MCS110	Spartalizumab	Breast and Pancreatic Cancer, Melanoma	NCT02807844	Overall Response: 27%
			Carboplatin, Gemcitabine	Advanced Triple-Negative Breast Cancer	NCT02435680	Clinical benefit: 29.4%
			Dabrafenib, Trametinib	Melanoma	NCT03455764	Not yet reported
		TPX-0022	Single agent	Advanced Solid Tumor	NCT03993873	Not yet reported
	Whole cell	Biphosphonates	Single agents	Primary Breast Cancer	NCT00127205	Overall survival: 92.4%
			Denosumab	Metastatic Breast Cancer	NCT00091832	Not yet reported
	Caspase 8	Trabectedin	Low-dose radiotherapy	Advanced/Metastatic Sarcomas	NCT05131386	Not yet reported
			Olaratumab	Advanced Soft-tissue Sarcoma	NCT03985722	Not yet reported
			Single agent	Malignant Pleural Mesothelioma	NCT02194231	Not yet reported
Inhibition of TAM recruitment	CCR2/CCL2	Carlumab	Single agent	Metastatic Castrate-Resistant Prostate Cancer	NCT00992186	Stable disease: 2.4%
			Single agent	Solid Tumors	NCT00537368	No results posted
			Chemotherapies	Solid Tumors	NCT01204996	Overall response: 38%
		Plozalizumab	Single agent	Bone Metastatic Solid Tumors	NCT01015560	Overall response: 14%
			ICIs	Advanced Melanoma	NCT02723006	Interrupted
		PF-04136309	Nab-paclitaxel, Gemcitabine	Metastatic Pancreatic Ductal Adenocarcinoma	NCT02732938	Objective response: 23%
			FOLFIRINOX	Pancreatic Neoplasms	NCT01413022	Objective response: 49%
		CCX872-B	Single agent	Pancreatic Adenocarcinoma	NCT02345408	Overall survival: 29%
	CCR2-CCR5	BMS-813160	Nivolumab, Chemotherapies	Pancreatic Ductal Adenocarcinoma	NCT03496662	Not yet reported
			Nivolumab, GVAX	Pancreatic Ductal Adenocarcinoma	NCT03767582	Not yet reported
			Chemotherapy, Nivolumab	Advanced Solid Tumors	NCT03184870	Not yet reported
			Nivolumab, BMS-986253	NSC Lung and Hepatocellular Carcinoma	NCT04123379	Not yet reported
	CCR5/CCL5	Maraviroc	Pembrolizumab	Metastatic Colorectal Cancer	NCT03274804	Disease Control: 5.3%
			Ipilimumab, Nivolumab	Metastatic Colorectal and Pancreatic Cancer	NCT04721301	Not yet reported
		Vicriviroc	Pembrolizumab	Advanced Colorectal Cancers	NCT03631407	No results posted
		Leronlimab	Single agent	Advanced Solid Tumors	NCT04504942	Not yet reported
			Single agent	Metastatic Triple-Negative Breast Carcinoma	NCT04313075	Not yet reported
			Carboplatin	Metastatic Triple-Negative Breast Carcinoma	NCT03838367	Not yet reported
	CXCR4/CXCL12	LY2510924	Sunitinib	Metastatic Renal Cell Carcinoma	NCT01391130	Insufficient Efficacy
			Carboplatin, Etoposide	Extensive Stage Small Cell Lung Carcinoma	NCT01439568	Insufficient Efficacy
			Durvalumab	Solid Tumors	NCT02737072	Interrupted
		Motixafortide	Cemiplimab, Chemotherapy	Pancreatic Adenocarcinoma	NCT04543071	Not yet reported
			Pembrolizumab	Metastatic Pancreatic Cancer	NCT02907099	Not yet reported
			Pembrolizumab, Onivyde^®^	Metastatic Pancreatic Cancer	NCT02826486	Disease Control: 77%
		Plerixafor	Cemiplimab	Metastatic Pancreatic Cancer	NCT04177810	Not yet reported
			Single agent	Pancreatic, Ovarian and CRC Cancers	NCT02179970	Stable disease: 57%
			Pembrolizumab	Head and Neck Cancer	NCT04058145	Interrupted

A highlight of the clinical benefit is reported only for the clinical trials with significative response to the treatments described. ‘Not yet reported’ results refer to either recruiting or non-recruiting active clinical trials. ‘No results posted’ refers to completed clinical trials with unavailable results.

**Table 2:** Summarized list of completed or active clinical trials targeting TAMs functions.

Strategy	Target	Drug Name	Combined Therapy	Disease	Clinical Trial
Reprogramming TAM polarization	TLRs	GSK1795091	GSK3174998, Pembrolizumab	Advanced Solid Tumors	NCT03447314
		Imiquimod	5-fluorouracil	Squamous Cell Carcinoma	NCT03370406
			Abraxane	Advanced Breast Cancer	NCT00821964
			Cyclophosphamide, Radiotherapy	Breast Cancer with Skin Metastases	NCT01421017
			Single agent	Breast Cancer with Skin Metastases	NCT00899574
		852A	Single agent	Unresectable Metastatic Cutaneous Melanoma	NCT00189332
			Single agent	Breast, Ovarian, Endometrial, Cervical Cancers	NCT00319748
		Resiquimod	gp100 and MAGE3 peptide vaccine	Melanoma	NCT00960752
		Motolimod	Durvalumab, Doxorubicin	Recurrent, Platinum-resistant Ovarian Cancer	NCT02431559
			Nivolumab	Head and Neck Cancer	NCT03906526
			Cetuximab	Metastatic Head and Neck Squamous Carcinoma	NCT01836029
		IMO-2055	FOLFIRI, Cetuximab	Colorectal Cancer	NCT00719199
			Erlotinib, Bevacizumab	Non-Small Cell Lung Cancer	NCT00633529
			Single agent	Clear Cell Renal Carcinoma	NCT00729053
		Tilsotolimod	Ipilimumab	Metastatic Melanoma	NCT03445533
			Single agent	Malignant Melanoma	NCT04126876
			Ipilimumab, Pembrolizumab	Metastatic Melanoma	NCT02644967
		CMP-001	Pembrolizumab	Head and Neck Squamous Cell Carcinoma	NCT04633278
			Nivolumab	Advanced Melanoma	NCT04698187
			Stereotactic body radiotherapy	Early-Stage Triple Negative Breast Cancer	NCT04807192
			Atezolizumab, Radiotherapy	Non-Small Cell Lung Cancer	NCT03438318
	CD40	Selicrelumab	Atezolizumab	Advanced Solid Tumors	NCT02304393
			Nab-paclitaxel, Gemcitabine	Pancreatic Cancer	NCT02588443
			Emactuzumab	Advanced Solid Tumors	NCT02760797
		SEA-CD40	Pembrolizumab, Carboplatin	Melanoma, Non-Small Cell Lung Cancer	NCT04993677
			Pembrolizumab, Gemcitabine	Advanced Solid Tumors	NCT02376699
		Sotigalimab	Doxorubicin	Soft Tissue Sarcoma	NCT03719430
			Pembrolizumab	Metastatic Melanoma	NCT02706353
			Single agent	Pediatric CNS Tumors	NCT03389802
		CP-870,893	Tremelimumab	Metastatic Melanoma	NCT01103635
			Paclitaxel + Carboplatin	Advanced Solid Tumors	NCT00607048
		CDX-1140	Pembrolizumab, Chemotherapy	Advanced Solid Tumors	NCT03329950
		ABBV-428	Nivolumab	Advanced Solid Tumors	NCT02955251
	PI3Kγ	Eganelisib	Nivolumab	Advanced Solid Tumors	NCT02637531
			Etrumadenant, doxorubicin, paclitaxel	Triple-Negative Breast and Ovarian Cancer	NCT03719326
			Nivolumab	Advanced Urothelial Carcinoma	NCT03980041
	HDACs	Tucidinostat	Tislelizumab	Metastatic Urothelial Carcinoma	NCT04562311
			Cisplatin	Metastatic Triple-negative Breast Cancer	NCT04192903
			Toripalimab	Advanced Cervical Cancer	NCT04651127
	STAT3	TTI-101	Single agent	Advanced Solid Tumors	NCT03195699
Re-activation of phagocytosis	CD47/SIRP1α	Magrolimab	Cetuximab	Advanced Solid Tumors	NCT02953782
			Avelumab	Ovarian Cancer	NCT03558139
			Dinutuximab	Neuroblastoma, Osteosarcoma	NCT04751383
			Docetaxel	Advanced Solid Tumors	NCT04827576
			Pactiltaxel, Nab-paclitaxel	Metastatic Triple-Negative Breast Cancer	NCT04958785
			Pembrolizumab, Chemotherapies	Head and Neck Squamous Cell Carcinoma	NCT04854499
		TTI-621	Rituximab, Nivolumab	Solid Tumors	NCT02663518
			ICIs, Radiation, T-Vec	Advanced Solid Tumors	NCT02890368
			Doxorubicin	Metastatic High-Grade Leiomyosarcoma	NCT04996004
		CC-95251	Rituximab, Cetuximab	Advanced Solid and Hematologic Cancer	NCT03783403
		CC-90002	Rituximab	Advanced Solid and Hematologic Cancer	NCT02367196
		STI-6643	Single agent	Advanced Solid Tumors	NCT04900519
Genetically engineering TAM	HER2-directed CAR-M	CT-0508	Single agent	HER2-overexpressing Solid Tumors	NCT04660929
